# NDUFA10‐Mediated ATP Reduction in Medial Prefrontal Cortex Exacerbates Burst Suppression in Aged Mice

**DOI:** 10.1111/cns.70453

**Published:** 2025-05-25

**Authors:** Huiwen Zhang, Panpan Fang, Gaolin Qiu, Dijia Wang, Jiqian Zhang, Zhilai Yang, Hu Liu, Qiying Shen, Xuesheng Liu

**Affiliations:** ^1^ Department of Anesthesiology, the First Affiliated Hospital of Anhui Medical University, Key Laboratory of Anesthesiology and Perioperative Medicine of Anhui Higher Education Institutes Anhui Medical University Hefei Anhui China

**Keywords:** adenosine triphosphate, aging, anesthesia, burst suppression, medial prefrontal cortex, NDUFA10, righting reflex recovery time

## Abstract

**Aims:**

Aging is associated with increased responsiveness to anesthesia‐induced burst suppression, which correlates with postoperative cognitive dysfunction and delirium. This study aims to investigate whether the enhanced burst suppression in aged mice under anesthesia is associated with a reduction in ATP levels within the medial prefrontal cortex (mPFC).

**Methods:**

EEG recordings were conducted to analyze the burst suppression, and in vivo fiber‐optic recording techniques were employed to monitor fluctuations in ATP levels within the mPFC during sevoflurane anesthesia. To elucidate the underlying mechanisms contributing to the observed variations in ATP levels in aged mice, mRNA sequencing was performed. Furthermore, site‐specific viral knockdown strategies were implemented to validate the mechanisms of action of key molecules.

**Results:**

We observed that sevoflurane anesthesia resulted in an increased burst suppression ratio, extended EEG suppression time, and reduced ATP levels in aged mice. Administration of ATP mitigated the anesthesia‐induced increase in EEG suppression time. RNA sequencing revealed that NDUFA10, an energy metabolism‐related gene, was down‐regulated in aged mice. Knockdown of NDUFA10 in mPFC increased burst suppression, whereas the administration of ATP attenuated these changes.

**Conclusions:**

NDUFA10‐driven ATP depletion in the mPFC prolongs sevoflurane‐induced burst suppression in aged mice, implicating energy metabolism regulation as a strategy to optimize geriatric anesthesia.

## Graphical abstract

Decreased expression of the mitochondrial complex I component protein NDUFA10 in the mPFC of aged mice leads to decreased ATP production, contributing to prolonged EEG suppression times during sevoflurane anesthesia and increased susceptibility to burst suppression.

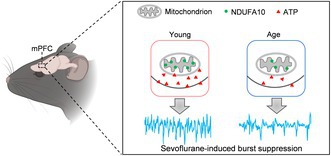

## 1 Introduction

As life expectancy increases globally, both the number and percentage of elderly individuals are rising, leading to an increasing demand for general anesthesia and surgery in this population. Although reversible, general anesthesia may result in postoperative adverse effects that impact quality of life and increase morbidity and mortality, particularly in older adults [1]. Burst suppression (BS) is a common neurophysiological phenomenon during the perioperative period, closely linked to morbidity and mortality. A recent study found that a reduction in intraoperative BS correlates with decreased 30‐day postoperative morbidity and mortality [2, 3]. Furthermore, aging is associated with prolonged BS, extended isoelectric periods, and reduced EEG amplitude in older patients under equivalent anesthetic drug concentrations [4, 5]. Aged mice exhibit increased sensitivity to anesthesia‐induced BS [6]. However, the mechanisms by which aging induces changes in EEG remain unclear.

BS consists of alternating periods of isoelectricity and active oscillations, typically observed during profound brain inactivity, such as deep anesthesia [7, 8, 9]. Several clinical studies have demonstrated a strong correlation between intraoperative BS and postoperative cognitive dysfunction [10, 11]. Intraoperative electroencephalographic monitoring is commonly employed to mitigate BS, thereby reducing the incidence of postoperative cognitive dysfunction. However, the mechanisms underlying BS remain unclear. A study of infantile epileptic encephalopathy, a condition characterized by BS, suggests that mitochondrial dysfunction is a primary contributor to its electroencephalographic manifestations [12, 13]. Additionally, pharmacologically induced BS may result from periodic ATP decreases and subsequent activation of K^+^‐dependent ATP channels [14]. Cerebral oxygen metabolism and ATP levels decrease during isoflurane anesthesia, with evidence suggesting that isoflurane‐induced BS is linked to reduced ATP consumption due to inhibition of synaptic activity [15].

ATP plays a critical role in brain energy metabolism and is closely tied to neuronal signaling [16]. Several studies have shown that mitochondrial dysfunction and reduced ATP production are linked to aging in the brain [17, 18]. Defective mitochondrial function has been shown to increase sensitivity to anesthetic agents in mice [19, 20]. Reducing energy levels and ATP through 2‐deoxy‐D‐glucose (2‐DG) enhances the effects of isoflurane anesthesia [21]. However, the effect of ATP on BS and anesthetic sensitivity remains inconclusive.

The prefrontal cortex (PFC), a brain region coordinating diverse higher‐order cognitive functions, comprises heterogeneous neuronal populations including projection neurons and interneurons [22]. Functional magnetic resonance imaging (fMRI) studies in rats have demonstrated widespread cortical engagement during anesthesia‐induced BS, suggesting that cortical neuronal activity is mechanistically linked to BS generation [23]. Furthermore, multimodal investigations integrating wide‐field fluorescence calcium imaging and EEG in animal models revealed robust correlations between PFC‐derived EEG signals and cortical neural dynamics during BS [24]. Notably, under equivalent burst suppression ratio (BSR) conditions across anesthetic agents, medial cortical regions exhibited significantly higher correlation coefficients compared to other areas, indicating a preferential association between medial cortical neuronal activity and BS [24]. Furthermore, healthy aging is characterized by an energy‐equivalent glucose deficit of approximately 8%, primarily localized to the PFC [16], suggesting a potential reduction in PFC ATP levels with aging. Based on these findings, we hypothesize that reduced ATP levels in the mPFC of aged mice increase suppression time.

To test this hypothesis, we monitored changes in EEG and ATP in the mPFC during anesthesia and reversed the increase in EEG suppression time and BSR in aged mice by microinjecting ATP into local brain regions. To elucidate the causes of reduced ATP levels in aged mice, we performed mRNA sequencing to analyze altered gene expression profiles related to energy metabolism. We also employed a viral knockdown approach to validate the hypothesis that reduced ATP levels contribute to the observed increase in EEG suppression time.

## 2 Methods and Materials

### 2.1 Animals

Male C57BL/6J mice (aged 6–8 weeks, 16–18 months) were obtained from Jiangsu Ji‐cui Yao‐kang Biotechnology Company (Nanjing, China). Under a 12‐h light/dark cycle (light from 8:00 am to 8:00 pm), the mice were grouped in captivity (with a maximum of five mice in each cage), and food and water were provided at will. The ambient temperature was maintained at 24°C ± 2°C, and the relative humidity was maintained at 55% ± 5%. The animal care and use in this study strictly abide by the institutional guidelines and government regulations, and all experiments are carried out according to the regulations approved by Anhui Medical University and Animal Research: Reporting of In Vivo Experiments (ARRIVE) guidelines, and the experimental approved number is LLSC20210071.

### 2.2 Surgical Procedures

The mice were anesthetized using pentobarbital sodium (20 mg/kg, intraperitoneally) and securely positioned in a stereotactic frame (RWD Life Science, China). To maintain body temperature, a warming pad was used, and eyes were protected with erythromycin ointment. An additional injection of lidocaine 0.5% (0.5 mL, subcutaneous injections) for analgesia was used to expose the skull. A midline scalp incision revealed the skull surface, which was then leveled before drilling holes using a dental drill. Virus was back filled into a pulled glass microelectrode, which was connected to a microsyringe pump (RWD Life Science, China). The injection coordinates of the mPFC were: AP: +1.8 mm; ML: ±0.35 mm; DV: −2.4 mm. The injection volumes of various viruses were 200 nL, with an infusion rate of 40 nL/min. After the injection, the syringe was left in place for 10 min to avoid vector reflux and then slowly pulled out.

For EEG/EMG recordings, EEG/EMG electrodes were implanted into the skull. Specifically, two stainless steel screws were placed in the cortex (recording electrode: AP, 1.3 mm posterior to bregma; ML, 1.5 mm lateral to bregma) and two others in the cerebellum (reference electrode). Electromyogram (EMG) electrodes were implanted into the bilateral neck extensor muscles.

For fiber photometry recordings and optogenetic manipulations, the optical fiber cannula was implanted above the mPFC (AP: +1.8 mm; ML: 0.35 mm; DV: −2.4 mm) [25], and cannulas were secured to the skull using dental adhesive resin cement.

For stereotaxic injection, bilateral guide cannulas (center‐to‐center distance 0.7 mm, RWD, Shenzhen, China) fitted with obturator and dust cap were implanted in the mouse mPFC, and cannulas were secured to the skull using dental adhesive resin cement. All mice were allowed to recover for at least 2 weeks before the experiments.

### 2.3 Viral Vectors and Chemicals

rAAV‐hSyn‐cyto‐iATPSnFR1.0, rAAV‐hSyn‐mcherry‐5'miR‐30ashRNA(mNdufa10)‐3′miR‐30a‐WPREs, rAAV‐hSyn‐mcherry‐5′miR‐30ashRNA(Scramble)‐3′miR‐30a‐WPREs were obtained from Brain Case (Braincase, Shenzhen, China). All viruses were subdivided into aliquots and stored at −80°C. ATP (Sigma‐Aldrich, #A7669, Burlington, MA, USA) was dissolved in phosphate‐buffered saline (1×) at 25 μmol/L. Apyrase (Sigma‐Aldrich, #A6132, Burlington, MA, USA) was dissolved in phosphate‐buffered saline (1×) at 300 U/mL [26].

### 2.4 In Vivo Fiber Photometry Recording

After 2 weeks of recovery and adaptation, the mice were recorded by fiber photometry. The fluorescence signals were recorded. LEDs (480 and 405 nm; Lumileds, China) were reflected by a dichroic mirror (Edmund optics, Barrington, United States) and focused through an objective lens (20×, NA0.4; Olympus, Japan). The laser power was adjusted to 40–70 μW to minimize bleaching. The fluorescence emission was bandpass filtered (87753; Edmund Optics, USA) and detected by a photomultiplier tube (H10721; Hamamatsu, Japan). An amplifier was used to convert the photomultiplier tube current output to voltage, which was further filtered through a low‐pass filter (35‐Hz cut‐off; Thinkertech, China). The analog voltage signals were digitalized at 100 Hz and recorded by fiber photometry software (Thinkertech). Before the experiment, the mice were placed in a cylinder containing oxygen 100% for 2 h for 3 consecutive days. After habituation, the mice were exposed to sevoflurane 3% in oxygen 100% for 20 min. Before exposure to sevoflurane, the mouse was connected to the optical fiber recording system and was free moving. When starting sevoflurane anesthesia, the time was recorded, and fiber recording was continued until the sevoflurane was stopped and the mice recovered. Data throughout the whole process of optical fiber recording were collected for subsequent data processing. After recording, the photometry data were exported to .mat files. Then, the values of fluorescence change (Δ*F*/*F*), defined as (*F* − *F*_0_)/*F*_0_, were calculated, where F_0_ is the baseline fluorescence signal averaged over a 100‐s control time.

### 2.5 Electroencephalogram Recording and Analysis

Mice were allowed a minimum of 14 days of recovery following surgery. Before the experiment, the mice were placed in a cylinder containing oxygen 100% for 2 h for 3 consecutive days. On the day of recording, the mice were acclimated first for 20 min in a recording box, where the temperature was kept at 25°C, and the mice were allowed to move around freely. After habituation, mice were exposed to sevoflurane 3% in oxygen 100% for 20 min. Before exposure to sevoflurane, the mouse was connected to the Medusa EEG/EMG recording system and was free moving. When starting sevoflurane anesthesia, the time was recorded, and EEG recording was continued until the sevoflurane was stopped and the mice recovered. All EEG signals were recorded at a sampling rate of 250 Hz using the Medusa EEG/EMG recording system (Bio‐Signal technologies, Jiangsu, China). The signals were filtered within a frequency range of 0.5–70 Hz. EEG data analysis was performed with MATLAB (version 2022a; The MathWorks Inc., Natick, MA, USA). EEG suppression was defined to have occurred when the wave amplitude was between −15 and 15 μV for > 0.2 s [27, 28, 29]. The cumulative suppression time, which was the sum of each single suppression time from the onset of sevoflurane anesthesia to its cessation, was calculated. BSR and relative power were analyzed by using artificial intelligence (AI)‐driven software Lunion Stage (https://www.luniondata.com, Shanghai, China).

### 2.6 Anesthesia Behavioral Testing

The duration of anesthesia recovery was quantified by measuring the recovery of righting reflex time, also referred to as the time to emergence, which serves as an indicator of recovery time. Briefly, the recovery of righting reflex time was defined when the mouse twisted from the supine position and placed all four feet on the floor within 30 s. In all experiments, the temperature of the mice was maintained by placing a 37°C heating pad beneath the chamber.

### 2.7 Histology and Microscopy

The mice were deeply anesthetized with sevoflurane and then transcardially perfused with ice‐cold phosphate‐buffered saline (0.1 M, pH 7.4). After substantial blood removal, 4% paraformaldehyde in phosphate‐buffered saline was used for perfusion. The brains were extracted and postfixed in 4% paraformaldehyde at 4°C overnight, followed by dehydration in 10%, 20%, and 30% sucrose solution overnight sequentially. The brains were then embedded in optimal cutting temperature compound (Sakura, Japan) and promptly frozen at −80°C, and 40 μm coronal sections encompassing the mPFC and optical fiber locations were obtained using a freezing microtome (MNT; SLEE, Germany). Brain sections were placed in phosphate‐buffered saline solution and subsequently pasted onto adhesion microscope slides. After the sections were dried and fixed, they were sealed with an anti‐fluorescence quencher containing DAPI (Beyotime, China, P0131). Finally, the tissue sections were imaged using a fluorescence microscope (cat. no. IX71; Olympus, Tokyo, Japan).

### 2.8 ATP Assay

ATP levels in the mPFC were determined using an Enhanced ATP Assay Kit (Beyotime, China, S0027) according to the instruction manual. Briefly, the mice were anesthetized deeply with sevoflurane and perfused transcranially with ice‐cold phosphate‐buffered saline (0.1 M, pH 7.4). Their mPFC tissues were harvested for the ATP assay. Lysate was added at a ratio of 200 μL of lysate per 20 mg of tissue and then homogenized using a tissue grinder. The lysed mPFC was centrifuged at 4°C and 12,000 *g* for 5 min, and the supernatant was collected. Prior to ATP detection, the assay solution was added to a black 96‐well plate and incubated for 5 min at room temperature. The supernatant was then added to the plate, mixed rapidly, and read within 30 min by the EnSpire multilabel reader (PerkinElmer, Waltham, Massachusetts, USA). Finally, calculate the total ATP content from the luminescence signal.

### 2.9 mRNA Sequencing Experimental Method

#### 2.9.1 RNA Isolation and Library Preparation

The mPFC harvested from mice in the young and aged groups was placed in liquid nitrogen for 1 h and then stored quickly in the freezer at −80°C for no longer than 1 week. mRNA isolation and library preparation, sequencing, and differential expression analysis were conducted by OE Biotech Co. Ltd. (Shanghai, China). Total RNA was extracted using the TRIzol reagent (Invitrogen, CA, USA) according to the manufacturer's protocol. RNA purity and quantification were evaluated using the NanoDrop 2000 spectrophotometer (Thermo Scientific, USA). RNA integrity was assessed using the Agilent 2100 Bioanalyzer (Agilent Technologies, Santa Clara, CA, USA). Then the libraries were constructed using VAHTS Universal V6 RNA‐seq Library Prep Kit according to the manufacturer's instructions.

#### 2.9.2 RNA Sequencing and Differentially Expressed Genes Analysis

The libraries were sequenced on an Illumina NovaSeq 6000 platform, and 150‐bp paired‐end reads (~50/sample) were generated. The raw reads were processed using fastp software, and low‐quality reads were removed, leaving approximately 40 clean reads. These reads were mapped to the reference genome using hierarchical indexing for spliced alignment of transcripts. For each gene, a fragments per kilobase of exon per million mapped fragments value was calculated, and read counts were obtained using HTSeq‐count. Principal component analysis was performed using R (v 3.2.0) to evaluate the biological duplication of samples. Differential expression analysis was performed using DESeq2 and the threshold for significantly differential expression of *p* < 0.05 and fold change > 1 or < 1. Hierarchical cluster analysis of differential gene expression was performed using R to demonstrate the expression patterns of genes in different groups and samples.

Based on the hypergeometric distribution, Gene Ontology (GO), Kyoto Encyclopedia of Genes and Genomes (KEGG) pathway, Reactome, and WikiPathways enrichment analyses of differential gene expression were performed to screen the significantly enriched terms using R. R was used to draw the column diagram and bubble diagram of the significant enrichment term. Gene Set Enrichment Analysis (GSEA) was performed using GSEA software. The analysis used a predefined gene set, and the genes were ranked according to the degree of differential expression in the two groups of samples. Then, we tested whether the predefined gene set was enriched at the top of the ranking list.

### 2.10 Western Blotting Analysis

The mice were anesthetized deeply with sevoflurane and perfused transcardially with ice‐cold phosphate‐buffered saline (0.1 M, pH 7.4). Their mPFC tissues were harvested for western blotting. Harvested tissues were homogenized with radio immunoprecipitation assay buffer (cat. no. BL504A; Biosharp, Hefei, China) with protease inhibitor (1:100, cat. no. BL507A; Biosharp) and phosphatase inhibitor (1:100, cat. no. BL615A; Biosharp) on ice. The homogenates were centrifuged at 4°C and 12,000 *g* for 10 min, and the supernatants were collected for western blotting. Protein sampling buffer (5×, cat. no. BL529B; Biosharp) was added to the samples at a ratio of 1:4, followed by heating for 15 min to denature the protein. Samples were put in equal volumes onto a 12% sodium dodecyl sulfate–polyacrylamide gel electrophoresis gels (Bio‐Rad Laboratories Inc., CA, USA) and electrophoresed at constant pressure before being transferred to polyvinylidene fluoride (PVDF) membranes (cat. no. IPVH00010; Sigma‐Aldrich). After the membranes were blocked with 5% nonfat milk for 1 h at room temperature, primary antibodies were applied for 16 h at 4°C. Then, the membrane was washed in tris buffered saline with tween‐20 (TBST) solution 4 times for 10 min each time. Finally, secondary antibodies were added, and the membranes were incubated for 1 h at room temperature and washed with TBST solution. The primary antibodies that were specifically used were the Ndufa10 antibody (1:1000, sc‐376,357, Santa Cruz Biotechnology) and the β‐Tubulin antibody (1:1000, cat. no. 2146; Cell Signaling Technology). Secondary goat anti‐rabbit antibody (1:10,000, cat. no. 7074; Cell Signaling Technology) and secondary horse anti‐mouse antibody (1:10,000, cat. no. 7076; Cell Signaling Technology) were used. Quantitative analysis of the protein bands was performed using a chemiluminescence instrument (Amersham Imager 600; GE Healthcare, PA, USA) after visualization with enhanced chemiluminescence detection reagent (cat. no. BL520A; Biosharp). Finally, protein levels were evaluated by quantifying the gray density of the western blot bands with ImageJ software (National Institutes of Health, Bethesda, MD, USA). The density of all samples was normalized to that of the internal control.

### 2.11 Quantitative Real‐Time PCR

Total RNA was extracted from mouse mPFC tissues using TRIzol reagent (Invitrogen, USA) according to the manufacturer's instructions. Then, the total RNA was reverse transcribed using a PrimeScript RT Reagent Kit with gDNA Eraser (TaKaRa, Japan). SYBR Premix Ex TaqTM II (TaKaRa, Japan) was used to amplify *NDUFA10* cDNA. *NDUFA10* gene expression was normalized by β‐actin expression. The data were analyzed using the $2^{-\Delta \Delta C_{t}}$ method, with β‐actin as an internal reference, to correct the *C*_t_ value of each sample and compare the expression of the target gene.

The sequences of the primers used for PCR were as follows: *NDUFA10* (mouse): forward, GAGGTTGCTGAGACTCGTCC, reverse, TATGAATTCGTCCCACGCGCT; *β‐actin* (mouse): forward, CAGCTTCTTTGCAGCTCCTT, reverse, CACGATGGAGGGGAATACAG.

### 2.12 Statistical Analysis

Statistical analyses and drawing were performed with GraphPad Prism 9.0 (GraphPad Software Inc., USA) and MATLAB R2022a (The MathWorks Inc., Natick, MA, USA). The data were presented as mean ± SEM. All data underwent normality testing using the Shapiro–Wilk test or Kolmogorov–Smirnov test. For comparisons between two groups of data, if the data exhibit a normal distribution, Student's *t*‐tests are used; if the data do not exhibit a normal distribution, unpaired Mann–Whitney tests are used. One‐way ANOVA or two‐way ANOVA are used to analyze data from more than two groups, followed by Tukey's multiple comparisons test. A *p*‐value < 0.05 was considered statistically significant.

## 3 Result

### 3.1 Prolonged Sevoflurane‐Induced BS in Aged Mice Correlates With mPFC ATP Reduction

**FIGURE 1:**
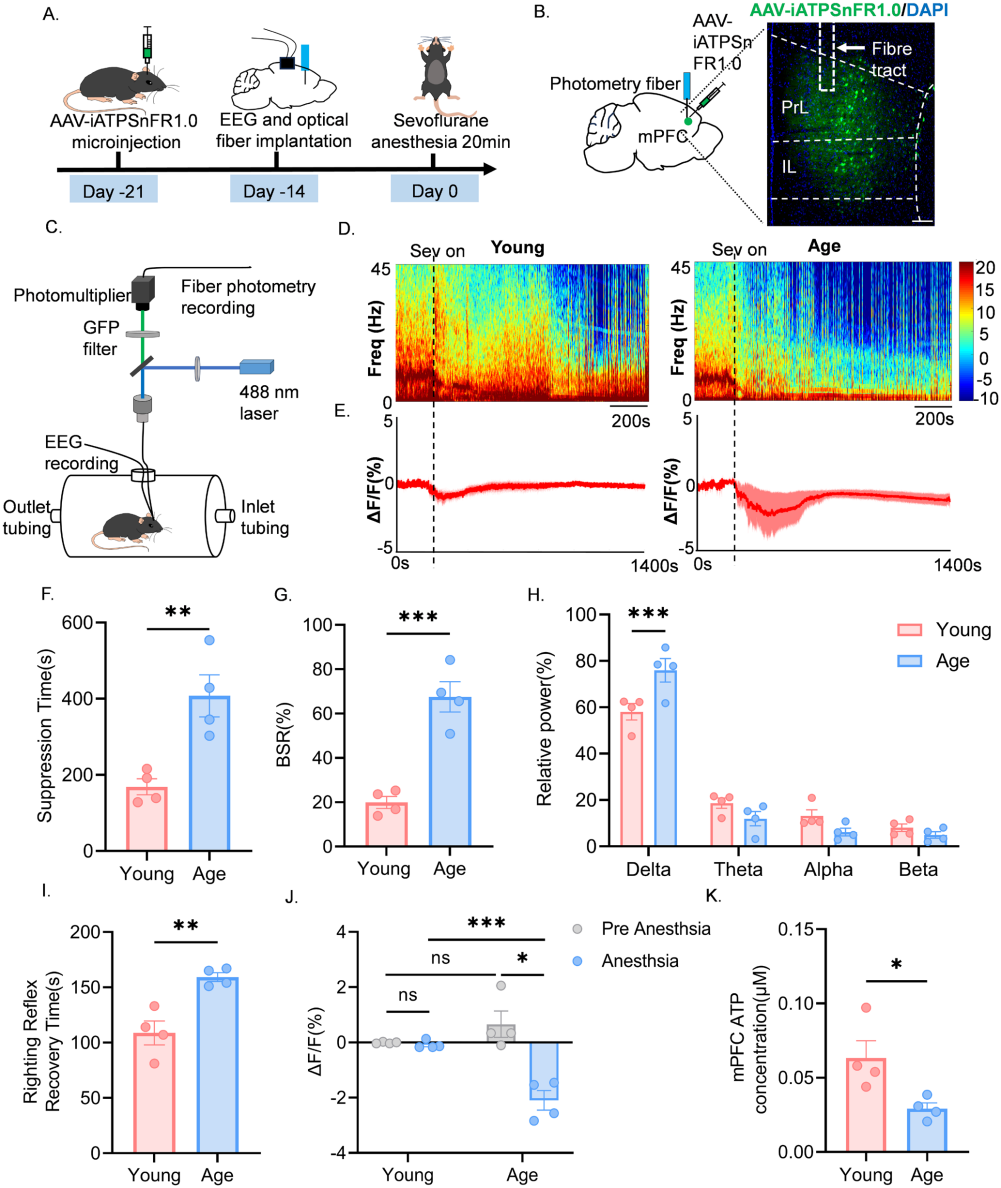
Prolonged sevoflurane‐induced BS in aged mice correlates with mPFC ATP reduction. (A) Schematic diagram of the experimental flow. (B) Schematic representation of AAV injection into the medial prefrontal cortex to express ATP1.0. Mice were implanted with fiber‐optic cannulae for fiber‐optic recordings (left). expression of ATP1.0 (green) in the medial prefrontal cortex of mice and fiber‐optic traces implanted above the medial prefrontal cortex (right). White dashed boxes indicate fiber‐optic traces, scale bar, 100 μm. (C) Schematic diagram of fiber‐optic and EEG recordings. (D) Representative EEG power spectrograms of young (left) and old (right) mice during sevoflurane 3% vol anesthesia, with black dashed lines and black arrows representing the onset of anesthesia. (E) Mean ATP transients (standard error of the mean [SEM]) during sevoflurane anesthesia (shaded area) in young (left) and old (right) mice (*n* = 4). (F) Suppression time for 20 min of sevoflurane 3% vol anesthesia, *n* = 4. (G) Burst suppression ratio for sevoflurane 3% vol anesthesia for 20 min, *n* = 4. (H) Relative power of delta band (0.5–4 Hz), theta band (4–8 Hz), alpha band (8–15 Hz), beta band (15–25 Hz) for 20 min of anesthesia with sevoflurane 3% vol, *n* = 4. (I) Time to recovery of the righting reflex at the end of sevoflurane 3% vol anesthesia, *n* = 4. (J) Quantification of changes in ATP signaling before and during anesthesia in young and old mice during sevoflurane 3% vol anesthesia, data are shown as mean (standard error [SEM]), *n* = 4. (K) ATP measurements showing ATP levels in isolated medial prefrontal cortex, *n* = 4. Data are expressed as mean ± SEM, *p < 0.05, **p < 0.01, ***p < 0.005, ns, no significance.

To investigate changes in suppression time and ATP levels in the mPFC during sevoflurane anesthesia in both aged and young mice, we injected rAAV‐hSyn‐cyto‐iATPSnFR1.0 into the mPFC and implanted EEG electrodes and optical fibers for simultaneous recording of EEG and ATP changes during anesthesia (Figure 1A–C). EEG spectrograms during anesthesia indicated that aged mice exhibited greater activity in the lower frequency bands while being significantly inhibited in the higher frequency bands; conversely, young mice showed the opposite pattern (Figure 1D). EEG analysis revealed that aged mice experienced significantly longer EEG suppression times compared to young mice (Figure 1F), higher BSR (Figure 1G), and greater δ‐wave power (Figure 1H). Furthermore, analysis of the recovery time of the righting reflex indicated that aged mice took significantly longer to regain consciousness than young mice (Figure 1I). In vivo monitoring of ATP changes in the mPFC showed a significant decrease in ATP levels in aged mice before and after anesthesia, whereas ATP levels in young mice remained unchanged. Additionally, ATP levels were lower after anesthesia in aged mice compared to young mice (Figure 1E,J). Measurements using an ATP assay kit revealed that the ATP concentration in the isolated mPFC of aged mice was significantly lower than that of young mice (Figure 1K). Consistent with clinical trial results, these data suggest that aged mice exhibit heightened sensitivity to anesthesia, as evidenced by increased EEG suppression time, higher BSR, and prolonged recovery time of the righting reflex at equivalent anesthetic concentrations, all accompanied by reduced ATP levels in the mPFC.

### 3.2 Augmented mPFC ATP Rescues EEG Suppression in Aged Mice

**FIGURE 2:**
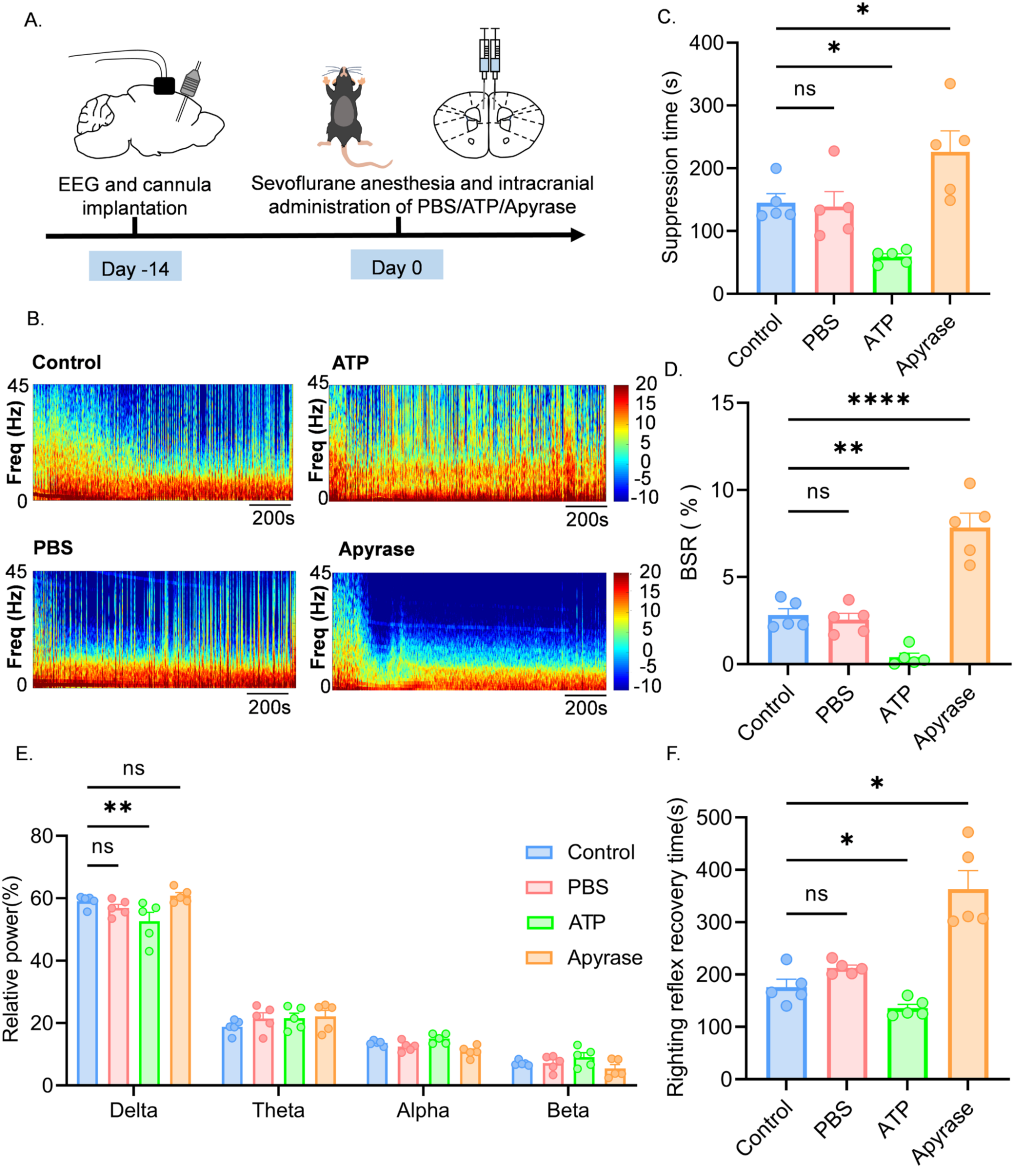
Augmented mPFC ATP rescues EEG suppression in aged mice. (A) Schematic diagram of the experimental flow. (B) Representative EEG power spectrograms during sevoflurane 3% vol anesthesia in the control, ATP, PBS, and Apyrase groups. (C) Suppression time for 20 min of sevoflurane 3% vol anesthesia (*n* = 5). (D) Burst suppression ratio for sevoflurane 3% vol anesthesia for 20 min, *n* = 5. (E) Relative power of delta band (0.5–4 Hz), theta band (4–8 Hz), alpha band (8–15 Hz), beta band (15–25 Hz) for 20 min of anesthesia with sevoflurane 3% vol, *n* = 5. (F) Recovery time of the righting reflex at the end of sevoflurane 3% vol anesthesia(*n* = 5). Data are expressed as mean ± SEM, *p < 0.05, ns, no significance.

To determine whether the increased EEG suppression time in aged mice was related to decreased ATP levels in the mPFC, we implanted bilateral injection cannulas in the mPFC of aged mice and administered PBS, ATP, or Apyrase simultaneously through the cannulas during anesthesia. A control group received no drugs, and EEG changes were monitored in all groups (Figure 2A). The EEG spectrograms indicated that mice exhibited activity in the high‐frequency band following ATP administration, while activity was observed in the low‐frequency band after Apyrase administration (Figure 2B). EEG analysis revealed a significant reduction in EEG suppression time in aged mice after ATP injection compared to the control group, with no change in the PBS group and an increase in the Apyrase group (Figure 2C). Consistent with EEG suppression time statistics, the BSR significantly decreased in the ATP group, remained unchanged in the PBS group, and significantly increased in the Apyrase group (Figure 2D). Furthermore, analysis of power across frequency bands indicated that δ‐wave power was significantly reduced in the ATP group, while δ‐wave power increased in the Apyrase group compared to the ATP group (Figure 2E). Statistical analysis of recovery times for the righting reflex showed that awakening time decreased following ATP injection, whereas awakening time increased in the Apyrase group, with no significant change in the PBS group (Figure 2F). These results suggest that increased EEG suppression time in aged mice is related to decreased ATP levels in the mPFC and that enhancing ATP levels can reduce EEG suppression time and lower BSR in aged mice.

### 3.3 Energy Metabolism Gene Downregulation in Aged Mice

**FIGURE 3:**
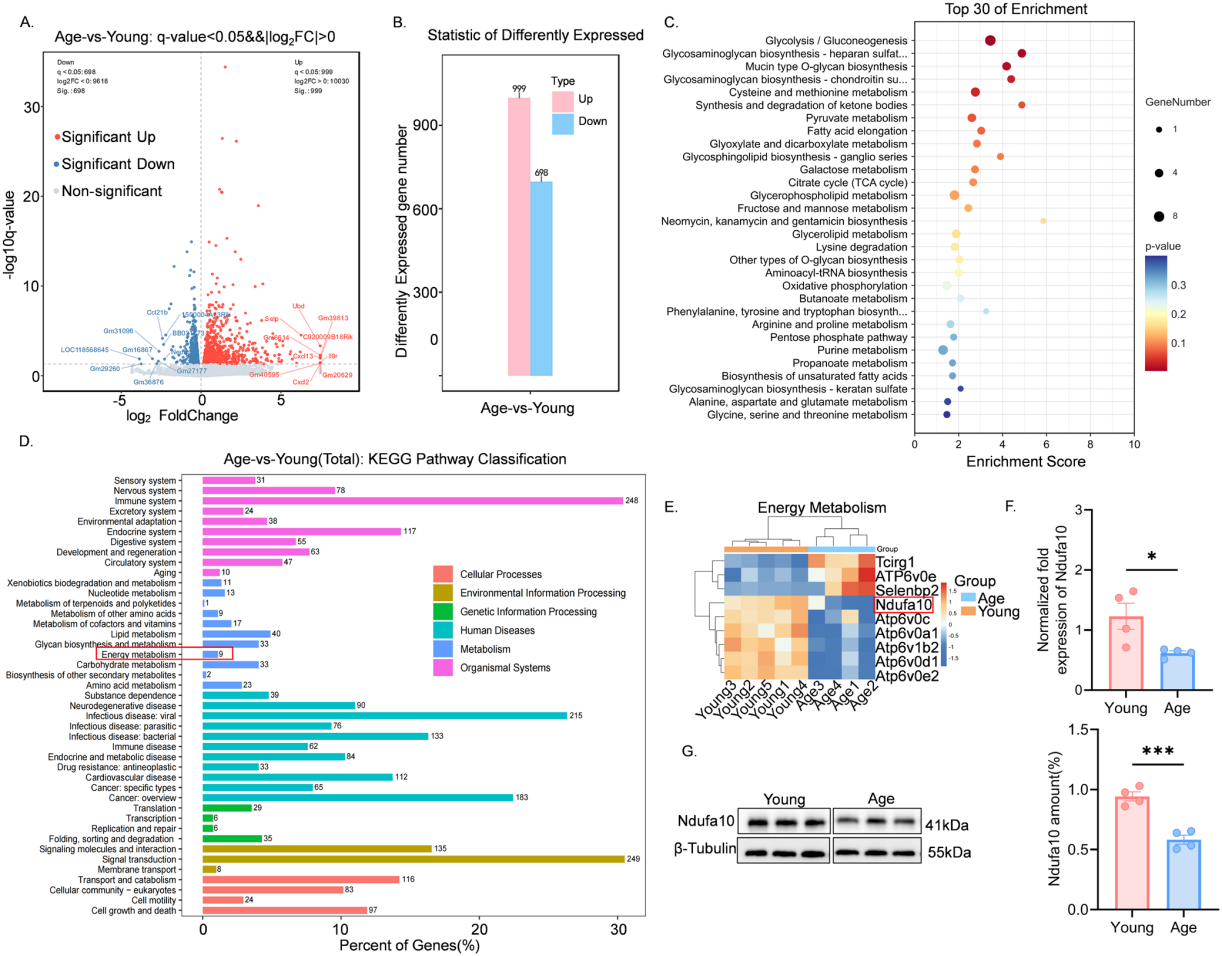
Energy metabolism gene downregulation in aged mice. (A) Volcano maps of differential genes in young and aged mice. Gray is non‐significantly different genes, red and blue are significantly different genes (young group, *n* = 5; aged group, *n* = 4). (B) Histogram of differentially expressed gene statistics. Up is the number of up‐regulated genes that are significantly different and down is the number of down‐regulated genes that are significantly different. (C) KEGG enrichment analysis of down‐regulated genes in both the young and aged groups top30 bubble plot. The horizontal axis Enrichment Score in the figure is the enrichment score, the bigger the bubble the greater number of differential protein‐coding genes are included in the entry, the color of the bubble changes from blue‐white‐yellow‐red, and the smaller the enrichment *p*‐value is, the greater the degree of significance is. (D) KEGG pathway distribution of differentially expressed genes. (E) Heat map of genes differentiating energy metabolism in the young and aged groups. (F) Gene expression of *Ndufa10* in medial prefrontal cortex detected by RT‐PCR (*n* = 4, Data are expressed as mean ± SEM, *p < 0.05). (G) Representative western blot bands of Ndufa10 protein expression (left) and quantitative analysis (right) (*n* = 4, Data are expressed as mean ± SEM, ***p < 0.005).

To identify the underlying cause of ATP reduction in the mPFC of aged mice, we conducted mRNA sequencing. The results revealed that 999 genes were up‐regulated and 698 genes were downregulated in the mPFC of aged mice compared to young mice (Figure 3A,B). KEGG enrichment analysis indicated that the downregulated genes were predominantly associated with metabolism‐related pathways, including the tricarboxylic acid (TCA) cycle and oxidative phosphorylation (Figure 3C). A total of nine differential genes were identified within the energy metabolism pathway (Figure 3D), of which three exhibited increased expression (*Tcirg1, Atp6v0e, Selenbp2*), whereas six demonstrated decreased expression (*Ndufa10, Atp6v0c, Atp6v0a1, Atp6v0b2, Atp6v0d1, Atp6v0e2*) (Figure 3E). The protein‐coding gene *NDUFA10* (NADH: ubiquinone oxidoreductase subunit A10) encodes a component of mitochondrial complex I and is associated with several pathways, including respiratory electron transfer, chemotaxis‐coupled ATP synthesis, and uncoupled protein heat production [30, 31]. Consistent with the mRNA sequencing results, decreased *Ndufa10* gene expression was confirmed in aged mice using real‐time fluorescence quantitative PCR and western blot analysis (Figure 3F,G). Previous studies have shown that disruption of mitochondrial complex I results in hypersensitivity to volatile anesthetic agents in humans, nematodes, and mice [32, 33, 34]. We therefore hypothesized that downregulation of the *Ndufa10* gene in the mPFC of aged mice leads to reduced ATP levels, which in turn results in heightened sensitivity to sevoflurane anesthesia‐induced BS.

### 3.4 mPFC Ndufa10 Knockdown Prolongs EEG Suppression Time

**FIGURE 4:**
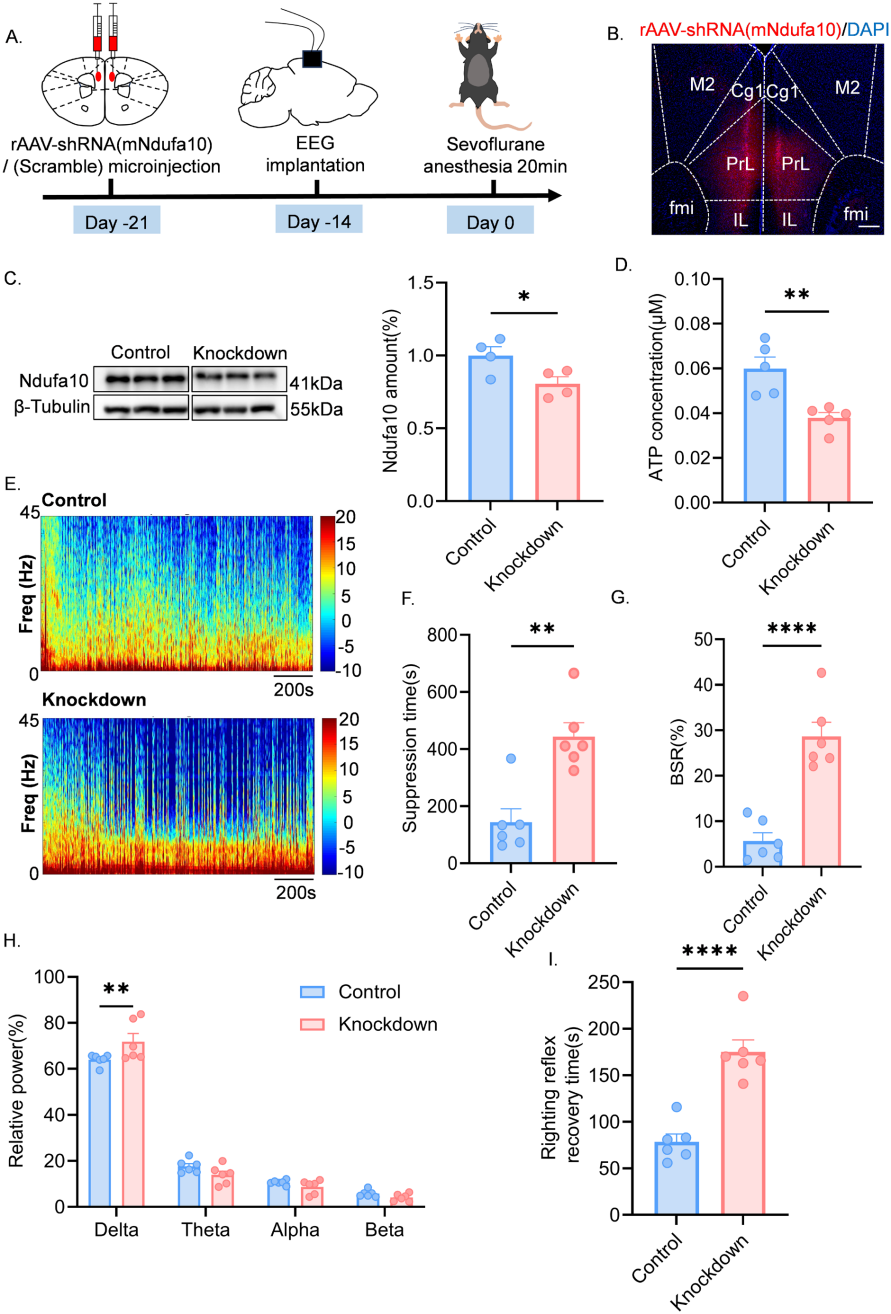
mPFC Ndufa10 knockdown prolongs EEG suppression time. (A) Schematic diagram of the experimental flow. (B) Schematic representation of rAAV‐shRNA (mNdufa10) (red) expression in medial prefrontal cortex (scale bar, 200 μm). (C) Representative western blot bands of Ndufa10 protein expression (left) and quantitative analysis (right) (*n* = 4). (D) ATP measurements showing ATP levels in isolated medial prefrontal cortex (*n* = 4). (E) Representative EEG power spectrograms during sevoflurane 3% vol anesthesia. (F) Suppression time for 20 min of sevoflurane 3% vol anesthesia in the control and knockdown groups (*n* = 6). (G) Burst suppression ratios for 20 min of sevoflurane 3% vol anesthesia in the control and knockdown groups, *n* = 6. (H) Relative power of delta band (0.5–4 Hz), theta band (4–8 Hz), alpha band (8–15 Hz), beta band (15–25 Hz) for 20 min of anesthesia with sevoflurane 3% vol in the control and knockdown groups, *n* = 6. (I) Recovery time of the righting reflex at the end of 20 min of sevoflurane 3% vol anesthesia in the control and knockdown groups (*n* = 6) Data are expressed as mean ± SEM, *p < 0.05, **p < 0.01, ****p < 0.001.

Subsequently, we performed bilateral knockdown of Ndufa10 in the mPFC using rAAV‐hSyn‐mcherry‐5′miR‐30a‐shRNA(mNdufa10)‐3′miR‐30a‐WPREs. Control subjects received injections of the control virus, rAAV‐hSyn‐mcherry‐5′miR‐30ashRNA (Scramble)‐3′miR‐30a‐WPREs, and were implanted with buried EEG electrodes (Figure 4A). The viral expression was confirmed to be complete after 3 weeks (Figure 4B). Western blot analysis demonstrated a significant reduction in Ndufa10 protein expression (Figure 4C). Measurement of ATP levels indicated that the Ndufa10‐knockdown group exhibited significantly lower ATP levels compared to the control group (Figure 4D). Mice were then anesthetized with 3% vol sevoflurane for 20 min, and EEG spectrograms revealed increased activity in the low‐frequency band for Ndufa10‐knockdown mice relative to controls (Figure 4E). EEG analysis indicated that the Ndufa10‐knockdown group had significantly longer EEG suppression times and higher BSR (Figure 4F,G). Concurrently, δ power was significantly elevated (Figure 4H). Furthermore, statistical analysis of righting reflex recovery times revealed that awakening time was prolonged in Ndufa10‐knockdown mice compared to controls (Figure 4I). These findings suggest that the knockdown of Ndufa10 in the mPFC of mice leads to a decrease in ATP levels, an increase in EEG suppression time, and heightened susceptibility to BS, analogous to changes observed in aged mice.

### 3.5 ATP Rescues Ndufa10 Knockdown‐Induced EEG Suppression Prolongation in mPFC

**FIGURE 5:**
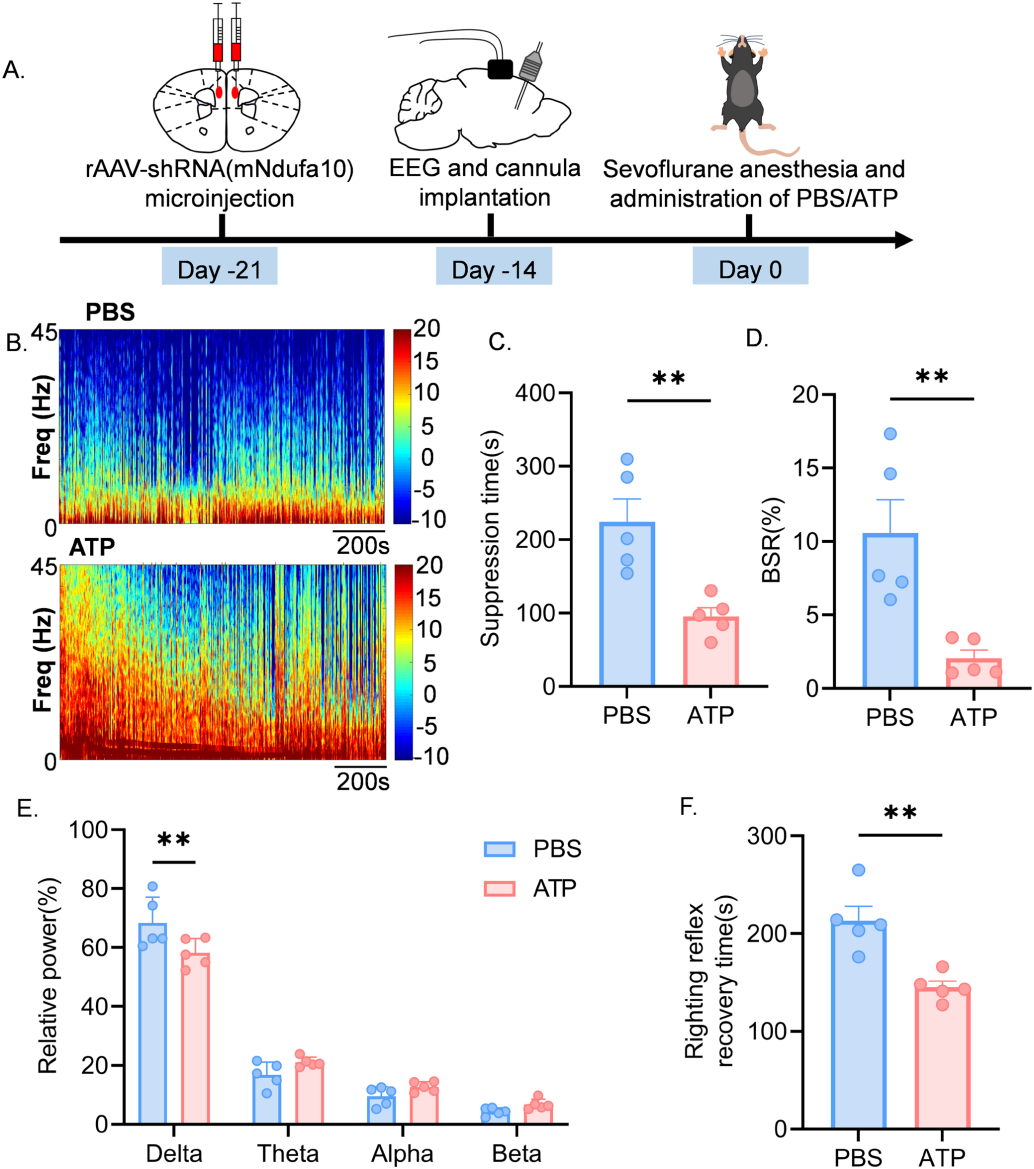
ATP rescues Ndufa10 knockdown‐induced EEG suppression prolongation in mPFC. (A) Schematic diagram of the experimental flow. (B) Representative EEG power spectrograms during sevoflurane 3% vol anesthesia. (C) Suppression time for 20 min of sevoflurane 3% vol anesthesia in the PBS and ATP groups (*n* = 5). (D) Burst suppression ratios for 20 min of sevoflurane 3% vol anesthesia in the control and knockdown groups, *n* = 5. (E) Relative power of delta band (0.5–4 Hz), theta band (4–8 Hz), alpha band (8–15 Hz), beta band (15–25 Hz) for 20 min of anesthesia with sevoflurane 3% vol in the ATP and PBS groups, *n* = 6. (F) Recovery time of righting reflex at the end of 20 min of sevoflurane 3% vol anesthesia in PBS and ATP groups (*n* = 5). Data are expressed as mean ± SEM, **p < 0.01.

To further elucidate the role of ATP in the mPFC, a bilateral injection cannula was implanted in Ndufa10‐knockdown mice, and ATP was administered during sevoflurane anesthesia at a concentration of 3% vol (Figure 5A). EEG spectrograms obtained during anesthesia revealed significant suppression of low‐frequency bands in the ATP group compared to the PBS group (Figure 5B). EEG analysis demonstrated a significant reduction in EEG suppression time in the ATP group (Figure 5C), as well as a notable decrease in BSR (Figure 5D) and δ‐wave power (Figure 5E). Additionally, statistical analysis of recovery times for the righting reflex indicated that awakening time was significantly shorter in ATP‐injected mice (Figure 5F). These findings suggest that ATP administration in the mPFC can effectively reduce EEG suppression time and lower BSR.

## 4 Discussion

**FIGURE 6:**
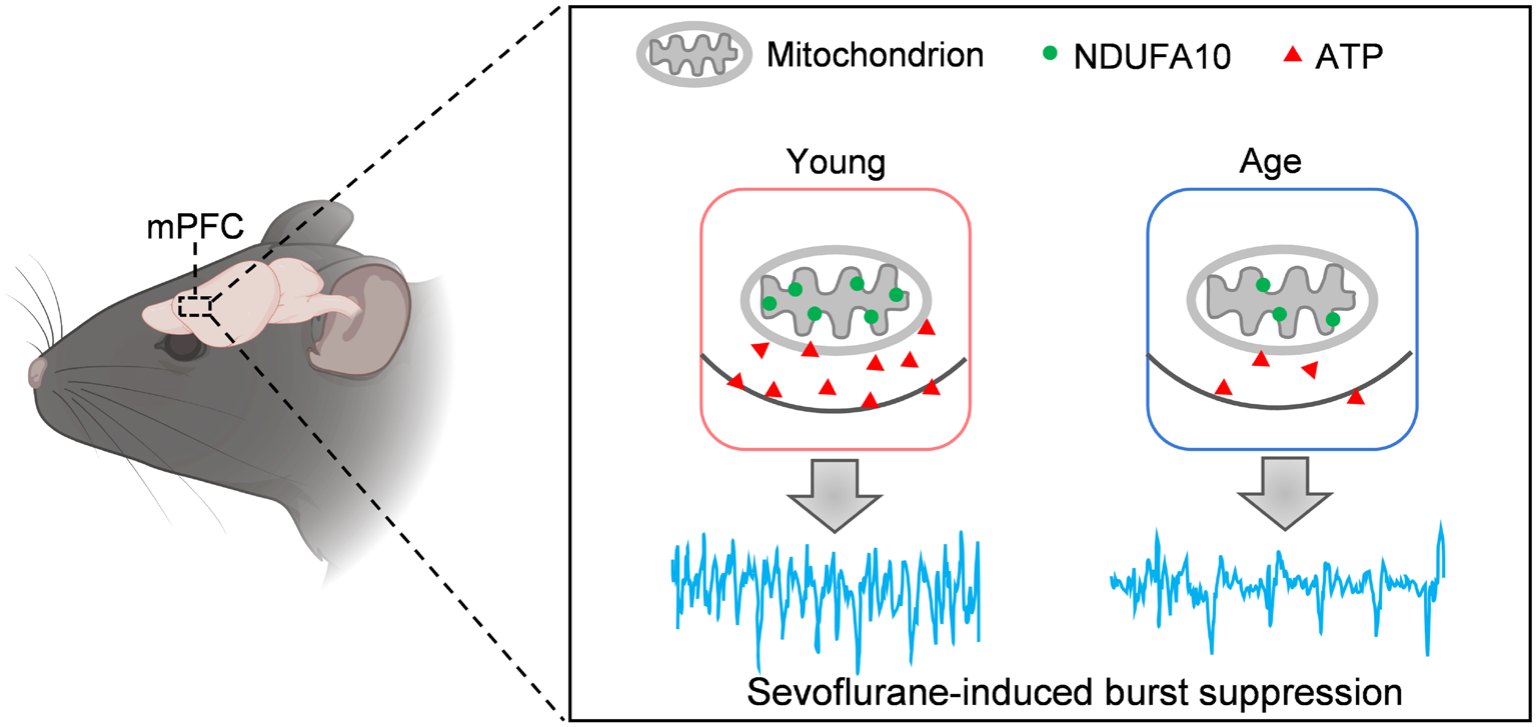
Schematic illustration of the mechanism of increased suppression time in aged mice. Decreased expression of the mitochondrial complex I component protein NDUFA10 in the mPFC of aged mice leads to decreased ATP production and causes increased suppression time during sevoflurane anesthesia. ATP, adenosine triphosphate; mPFC, medial prefrontal cortex.

This study presents the first simultaneous monitoring of in vivo ATP and EEG changes during anesthesia using fiber‐optic and EEG recordings. Our findings indicate that the increased suppression duration observed in aged mice is attributable to decreased ATP levels. Additionally, mRNA sequencing revealed that diminished expression of the *NDUFA10* gene contributes to the reduced ATP and facilitates the easier induction of BS in aged mice (Figure 6).

BSR is a reliable parameter for assessing the depth of anesthesia in clinical settings [35]. It is also hypothesized that intraoperative BS correlates with postoperative delirium in elderly patients [36, 37]. However, the mechanisms underlying BS remain unclear. Ching et al. employed a mathematical model to elucidate these mechanisms, demonstrating that BS arises from the interaction between neuronal dynamics and brain metabolism. They further showed that periodic alterations in BS are linked to ATP production and utilization [14]. Our results indicate that, during anesthesia, the onset of BS is accompanied by a decrease in ATP levels in aged mice. This finding further validates the metabolic model of BS, as modulation of ATP levels influences both suppression time and BSR. Although general anesthetic agents are known to induce mitochondrial dysfunction and reduce ATP levels [38, 39], our findings suggest that, in young mice, ATP levels do not significantly decline during anesthesia, and EEG suppression times are shorter. We speculate that this may be due to young mice having intact or only mildly impaired energy metabolism, combined with the use of sevoflurane, which exerts minimal effects on mitochondria and energy metabolism [40]. Consequently, young mice may effectively compensate for the anesthesia‐induced decrease in ATP.

The aging process has been shown to increase anesthetic sensitivity in both humans and animals, with a corresponding decrease in the required anesthetic dose as age progresses [41]. EEG analyses reveal that while power and amplitude decrease with age, BS increases [42, 43]. Consistent with these findings, our data suggest that aged mice undergoing sevoflurane anesthesia exhibit prolonged EEG suppression times, are more likely to enter BS, and have greater difficulty recovering from anesthesia. This underscores the necessity of considering age‐related changes when using EEG to guide anesthesia in clinical practice to individualize anesthetic dosages. Furthermore, mitochondrial function and energy levels play significant roles in anesthetic sensitivity [32, 33, 44]. In a related study, Wang et al. enhanced isoflurane anesthesia by reducing brain ATP levels with 2‐DG, finding that coenzyme Q10 could reverse the effects of 2‐DG on anesthesia by rescuing ATP depletion [21]. Notably, our study utilized fiber‐optic recordings to monitor ATP level changes in the mPFC during anesthesia, revealing a similar reduction in energy metabolism and ATP levels in aged mice. This suggests that increased anesthetic sensitivity in older subjects may be linked to diminished energy metabolism and lower ATP levels.

In clinical settings, the study explores the heightened sensitivity to sevoflurane anesthesia observed in patients with mitochondrial Complex I disorder [33, 45]. The *NDUFS4* gene, which encodes a subunit of mitochondrial complex I, has been implicated in hypersensitivity to volatile anesthetics following knockout in mice [46, 47, 48]. Compared to baseline cortical EEG, power was preserved in the lower frequency bands but decreased in the higher frequency bands [49]. Similar to *NDUFS4*, the *NDUFA10* gene encodes a protein integral to mitochondrial complex I. Through transcriptome sequencing, we identified a significant reduction in NDUFA10 expression in aged mice, which may account for the decreased ATP levels and heightened sensitivity to sevoflurane anesthesia. By knocking down NDUFA10 in the mPFC of young mice, we observed decreased ATP levels, longer EEG suppression times, increased low‐frequency band power, and prolonged recovery from anesthesia. These results indicate that reduced NDUFA10 expression contributes to increased anesthetic sensitivity in aged mice.

The mPFC is crucial for generating EEG rhythms, and occipital EEG power is positively correlated with regional cerebral blood flow (rCBF) in this region [50]. Neuronal activity in the medial cortex is strongly associated with BS [24]. Our correlation analysis between ATP levels and BS in the mPFC, supported by local ATP injections, suggests a causal relationship. Clinical studies indicate a positive correlation between BS duration and postoperative delirium [10], though whether BS can serve as a predictor of postoperative delirium remains contentious. In this study, we demonstrate that reduced ATP in the mPFC leads to extended EEG suppression times. Given that the mPFC regulates various cognitive functions such as working memory, decision‐making, and attention [51, 52], and mediates memory deficits in mice with postoperative cognitive dysfunction [53, 54], we propose that the mPFC serves as a hub linking BS and postoperative cognitive dysfunction. Future research will aim to elucidate the specific neuromolecular mechanisms by which ATP levels in the mPFC affect BS and its modulation of postoperative cognitive function, providing evidence for utilizing BS to guide anesthesia and reduce the incidence of postoperative delirium.

This study has certain limitations. First, female subjects demonstrate greater resistance to volatile anesthetics in both humans and mice [55]. Male mice exhibit higher anesthetic sensitivity at equivalent anesthetic concentrations, with their sensitivity bidirectionally modulated by testosterone, and there are noted sex differences in EEG responses to sevoflurane anesthesia. At the same concentration of sevoflurane, female mice exhibited delayed BS onset and lower BSR compared to males. Analysis of the relative power in the delta band during anesthesia induction and emergence revealed more pronounced δ power in male mice at 30‐s intervals [56]. However, we exclusively used male mice in this study, necessitating further verification to determine if increased suppression times in aged females correlate with ATP levels. Second, different anesthetic agents acting on various receptors produce distinct EEG changes; sevoflurane‐induced BS exhibited a significantly longer duration and higher power compared to propofol‐induced BS [57]. Additionally, our study specifically investigated the effects of sevoflurane on ATP levels in mice, while whether other anesthetic agents modulate ATP levels via NDUFA10 (a mitochondrial complex I subunit) remains unexplored. Notably, general anesthetics, including isoflurane, sevoflurane, pentobarbital, and propofol, have been shown to inhibit mitochondrial Complex I or II of the electron transport chain, thereby reducing ATP production [38, 39]. The magnitude and direction of sensitivity changes to anesthetic agents in both humans and mice with mitochondrial Complex I dysfunction are heterogeneous across drug classes. Notably, volatile anesthetics elicit the highest sensitivity, while hypersensitivity to propofol is less pronounced compared to volatile agents [34]. Therefore, we need to further investigate whether BS induced by other anesthetics (e.g., propofol) is associated with ATP levels.

In summary, our study demonstrates that decreased NDUFA10 expression in the mPFC of aged mice results in reduced ATP levels, contributing to prolonged EEG suppression times during sevoflurane anesthesia and increased susceptibility to BS. These findings have significant implications for the clinical application of BS in guiding anesthesia in elderly patients and provide support for the metabolic hypothesis of BS.

## Author Contributions

H.Z. and PF.: experimental design, data collection, data analysis and interpretation, and article writing; G.Q., D.W., J.Z., Z.Y., H.L., and Q.S.: data analysis and article writing; X.L.: conception and design, financial support, article writing, and final approval of the article. All authors have read and agreed to the published version of the manuscript.

## Conflicts of Interest

The authors declare no conflicts of interest.

## Supporting information


Data S1

## Data Availability

The data that support the findings of this study are available on request from the corresponding author. The data are not publicly available due to privacy or ethical restrictions.
