# Supplementary figures and images for: NDUFA10‐Mediated ATP Reduction in Medial Prefrontal Cortex Exacerbates Burst Suppression in Aged Mice
Source: CNS Neurosci Ther. 2025 May 25;31(5):e70453. doi: 10.1111/cns.70453 (PMC12104567; doi:10.1111/cns.70453)

Full unedited blot for Figure 3G

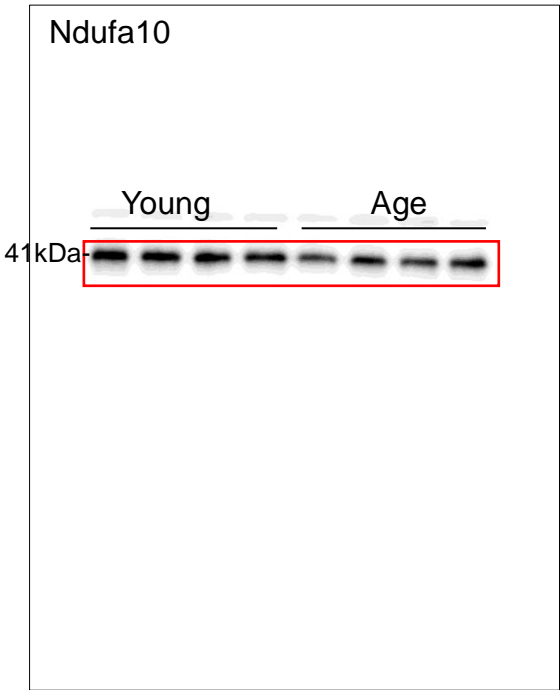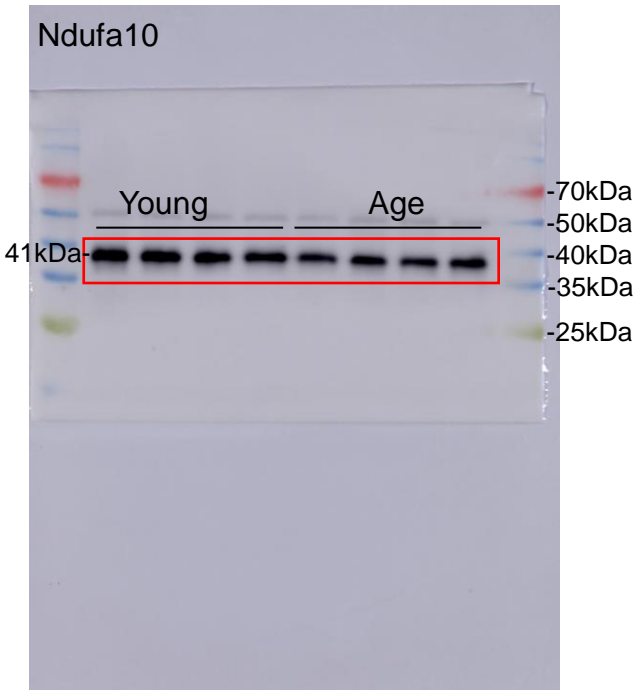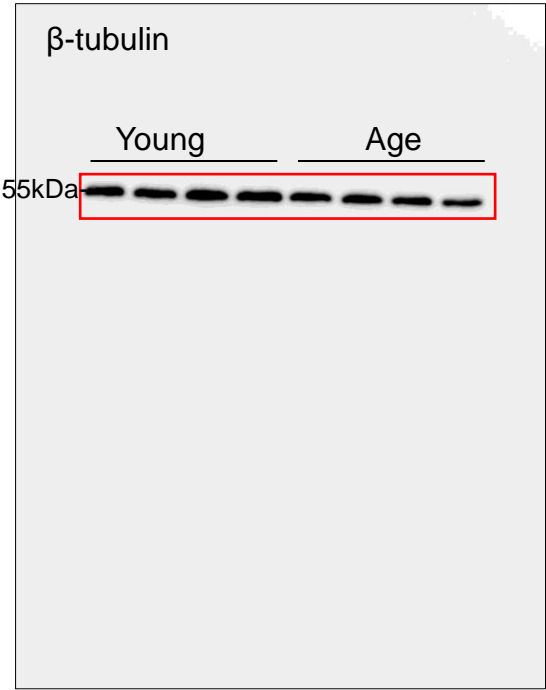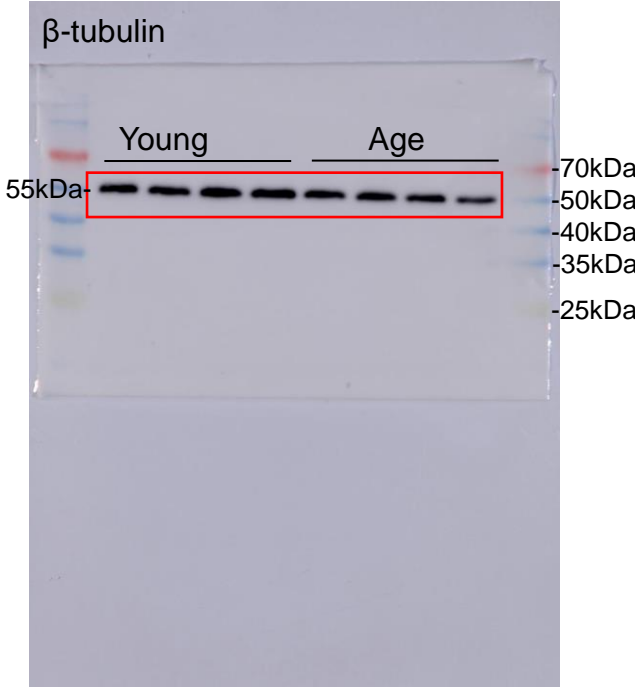

Full unedited blot for Figure 4C

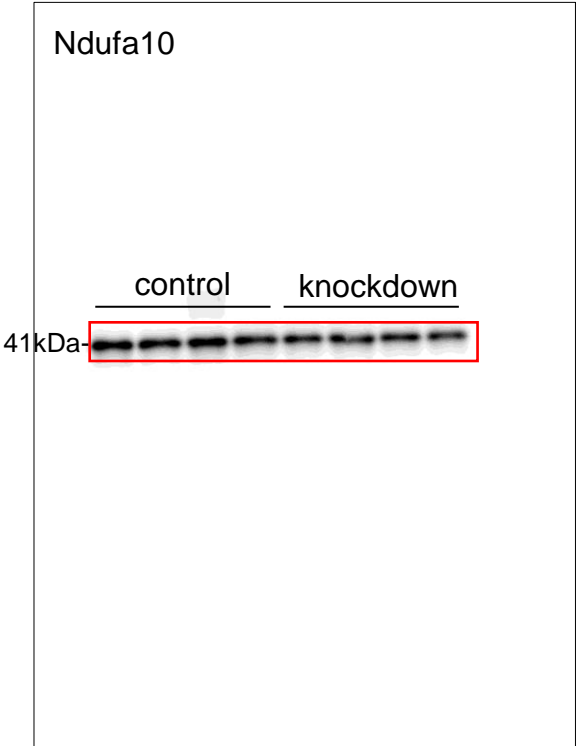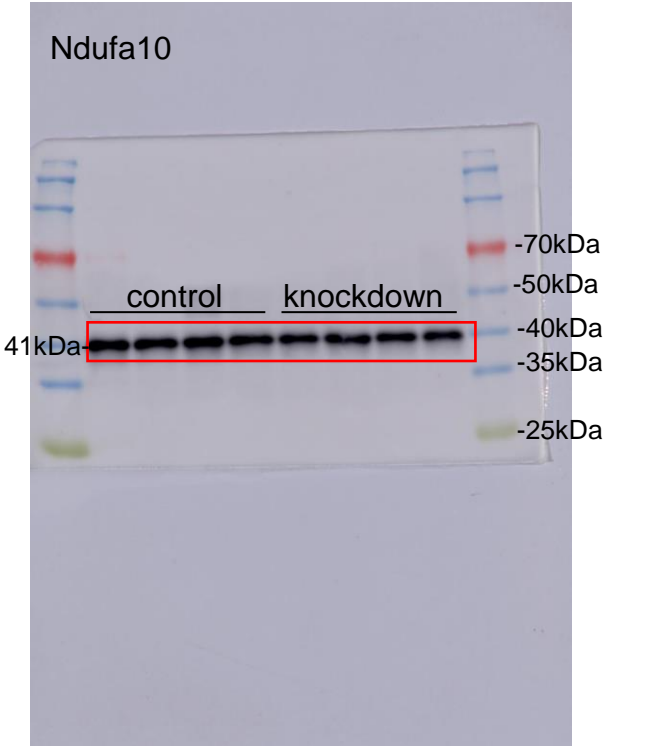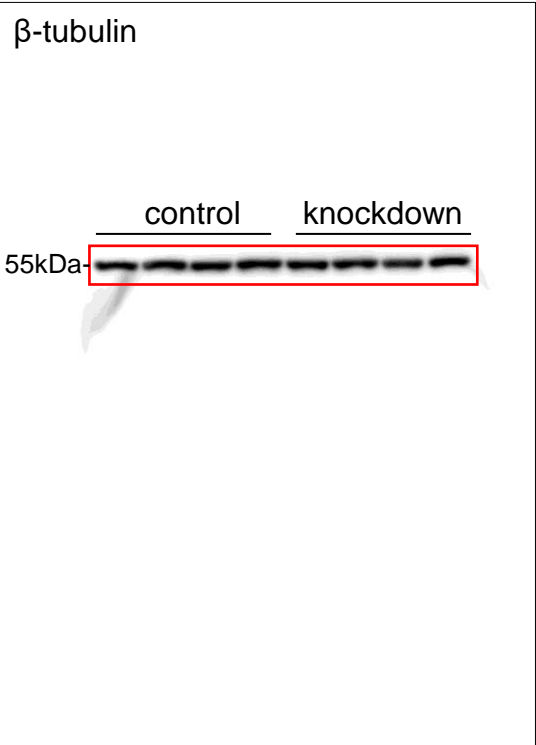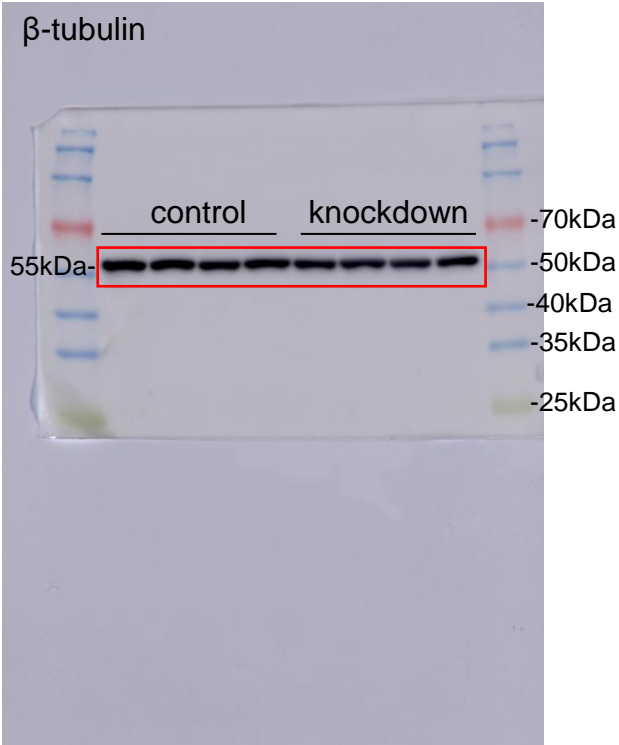

Supplement: Supplementary file 1 — Data S1 [file CNS-31-e70453-s001.pdf]
